# Understanding the complex interplay of barriers to physical activity amongst black and minority ethnic groups in the United Kingdom: a qualitative synthesis using meta-ethnography

**DOI:** 10.1186/s12889-015-1893-0

**Published:** 2015-07-12

**Authors:** Sejlo A. Koshoedo, Virginia A. Paul-Ebhohimhen, Ruth G. Jepson, Margaret C. Watson

**Affiliations:** Centre of Academic Primary Care, University of Aberdeen Polwarth Building, Foresterhill, Aberdeen, AB25 2ZD UK; NHS Highlands Occupational Health Service Osprey House, Raigmore Avenue, Inverness, IV2 3DZ UK; Scottish Collaboration for Public Health Research and Policy (SCPHRP), University of Edinburgh, 20 West Richmond Street, Edinburgh, EH8 9DX UK

**Keywords:** Ethnic groups, Physical activity, Culture, Lifestyle, Migrants, Minority health, Systematic review, Black population, Meta-ethnography

## Abstract

**Background:**

To conduct a meta-ethnographic analysis of qualitative studies to identify barriers to Black and Minority Ethnic (BME) individuals engaging in physical activity in the UK context.

**Methods:**

A qualitative synthesis using meta-ethnographic methods to synthesis studies of barriers to engaging in physical activity among BME groups in the UK. A comprehensive search strategy of multiple databases was employed to identify qualitative research studies published up to October 2012. The eleven searched databases included ASSIA, MEDLINE, EMBASE, CINAHL, Health Technology Assessment (HTA), NHS Scotland Library, Physical Activity Health Alliance (PAHA), PsyINFO, Social Services Abstract, Sport discuss and Web of Science. The Noblit and Hare’s meta-ethnographic approach was undertaken to develop an inductive and interpretive form of knowledge synthesis.

**Results:**

Fourteen papers met the inclusion criteria. The synthesis indicated that barriers to physical activity among BME individuals were influenced by four main concepts: perceptions; cultural expectations; personal barriers; and factors limiting access to facilities. BME individuals had different understandings of physical activity were influenced by migration history, experiences, cultural and health beliefs. This in turn may have a disempowering effect on BME individuals in terms of adopting or maintaining physical activity. These barriers to physical activity were explained at a higher conceptual level by a socio-ecological model. The social construct ‘individual perception and understanding of physical activity’ was particularly relevant to theoretical models and interventions.

**Conclusion:**

Interventions to promote engagement with physical activity need to address perceptions of this behaviour. The elicited concepts and contexts could be used to enhance the development of tailored effective health promotion interventions for BME individuals.

**Electronic supplementary material:**

The online version of this article (doi:10.1186/s12889-015-1893-0) contains supplementary material, which is available to authorized users.

## Background

Inactive lifestyles contribute to increasing social and health inequalities among Black and Minority Ethnic (BME) groups [1]. Physical activity levels have been reported low among BME groups compared with the general population in the UK [2–4]. The benefits of physical activity in terms of health outcomes outweigh its disadvantages. Physical activity reduces mortality and the burden of major non-communicable diseases including diabetes mellitus, cancer, obesity, hypertension, stroke, osteoporosis, osteoarthritis, and depression [5]. Ethnicity influences health status and behaviour BME individuals have a higher prevalence of non-communicable disease compared with majority population, especially cardiovascular disease, stroke, hypertension [6, 7], and diabetes [8]. There is a need to understand the factors that limit BME individuals’ participation in physical activity so that health and social consequences can be addressed. In developing an intervention, the relevance of Medical Research Council (MRC) framework for complex interventions is acknowledged to provide several dimensions of complexity, interactions and levels between the components of interventions [9].

The UK’s BME population or non-majority population is small but growing, as evidenced from the Census where a rise from 7.9 % to 14 % between 2001 and 2011 was noted in England and Wales [10]. In Scotland, a rise from 2 % to 3.7 % of the population was described as non-majority population between 2001 and 2011 Census [11]. The BME population in the UK is diverse and constitutes individuals from Africa and Asia (including Indian, Pakistani or Bangladeshi). Hence non-Black population may be considered as an ethnic minority group. A quarter of minority ethnic people described themselves in the Census as Black, which includes Black Caribbean, Black African or Other Black [10]. The health risk in BME groups is reflected in various non-communicable diseases. For example, the risk of stroke in a South Asian individual is 1.5 fold greater compared with majority populations in the UK [6, 12, 13], and the risk in Afro-Caribbeans is up to 2.5 fold greater than the general population [14]. Furthermore, the burden of obesity in the UK is notable among Black African women (38.2 %) and Black Caribbean men (25.2 %) compared with the general population (23.2 % and 22.7 %, respectively) [15]. This may be closely related to the risk of developing Type 2 Diabetes. The risk of Type 2 Diabetes in BME individuals is up to six times greater in people of South Asian descent and up to three times more common among people of African and African-Caribbean origin than the majority population [8, 12, 13].

In the United Kingdom (UK), tackling BME individuals’ low participation in physical activity is central to Government attempts to reduce health inequalities and health risk of non-communicable diseases. Popular UK Government policies include ‘Let’s Make Scotland More Active’ [16], ‘Equally Well’ [17], ‘Tackling Health Inequalities’ [18], ‘Choosing activity’ [19], ‘At least five a week’ [20], ‘Build more cohesive and active communities’ [21], and ‘Promote better health and wellbeing for all [22]. Developing these and further policies requires a solid base of UK evidence on BME groups’ experiences of physical activity. The information about experiences and practices of BME groups is crucial to informing interventions and policies to promote physical activity. Although physical activity is used interchangeably with concepts such as ‘exercise’ , ‘sport’ and ‘physical fitness’ [23], this review focused on range of behaviour resulting from daily activities. According to World Health Organisation (WHO), physical activity is defined ‘as any bodily movement produced by skeletal muscles that require energy expenditure above the resting level’ [24]. Hence, for the purpose of this review, physical activity encompasses range of behaviour which includes leisure and non-leisure activities in daily life, occupational or household tasks engaged in with the aim of improving fitness and health [2].

In the last decade, the use of meta-ethnography has gained attention among various healthcare disciplines and the method is considered suitable for reviews that involve perceptions of disease and high risk behaviour [25]. The systematic approach that identifies themes and relationships between studies distinguishes meta-ethnography from conventional narrative reviews. Meta-ethnography produces higher level interpretations, greater explanatory power and generates theory from multiple studies compared with traditional narrative reviews [26–28]. Meta-ethnographic synthesis goes beyond the summary of studies and can be used for conceptual development to foster theoretical advancement, and to inform practice and policy [28]. This study aimed to address the main research question of how meta-ethnography can add to previous narrative reviews by deriving new conceptual understanding of barriers to engaging in physical activity among BME individuals and their interrelationship through a meta-ethnography synthesis. In addition study addressed how resulting over-arching concepts produce higher level interpretations with implications for practice, policy and further research.

## Methods

A systematic review of qualitative studies was conducted using meta-ethnographic synthesis. The review was conducted and reported to comply with the ‘Enhancing transparency in reporting the synthesis of qualitative research (ENTREQ) statement [29].

### Data sources and search strategy

**Fig. 1:**
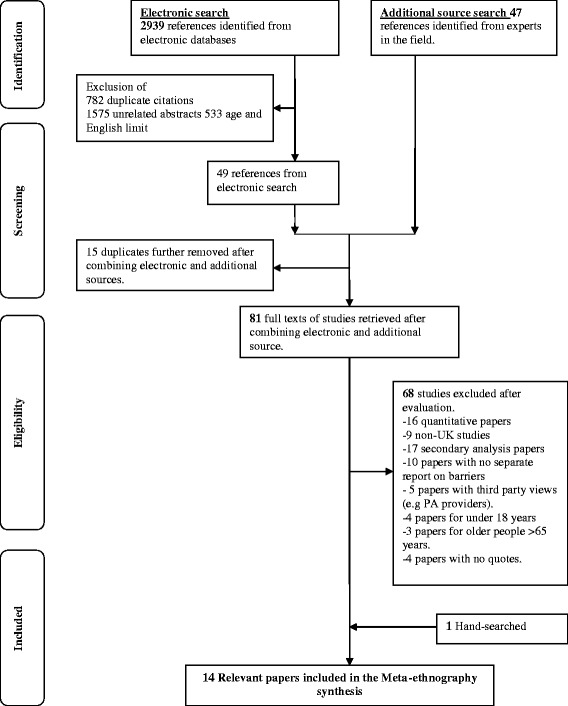
PRISMA Flow Diagram of Study Selection Process. A flow diagram describes the stages and results of identification, screening, eligibility and inclusion of qualitative studies

The search was conducted using a database of experts in the field (NHS Health Scotland) and 11 electronic bibliographic databases: ASSIA, MEDLINE, EMBASE, CINAHL, Health Technology Assessment (HTA), NHS Scotland Library, Physical Activity Health Alliance (PAHA), PsyINFO, Social Services Abstract, Sport discuss and Web of Science. The search strategy was adapted from previous work [30]. Therefore, a systematic search was conducted from January 1990 to October 2012. Government web pages of the NHS Scotland Library, Health Technology Assessment and Physical Activity and Health Alliance were also searched to identify studies not identified from generic databases but which reflected data used to guide government policies and initiatives. Figure 1 illustrates the search process. Synonyms for the broad terms of ‘Physical activity’ , ‘BME groups’ and ‘UK’ were combined to identify qualitative studies that explored experiences, perceptions, attitudes, practice and barriers related to physical activity. Bibliographies of identified additional studies were hand searched to identify papers that met the eligibility criteria for this systematic review.

### Inclusion and exclusion criteria

The studies were independently screened by two reviewers S.K and V.P-E in relation to the inclusion and exclusion criteria (Table 1). Studies were restricted those that included BME individuals within ages 18 to 65 years. The eligibility criteria considered studies targeting participants that reflected BME groups in the UK only, in consideration of the heterogeneity of factors that determines agreed minority groups criteria in different country contexts. For example, studies from the US commonly include ethnic groups such as Hispanics and Latino Americans who are likely to have barriers of physical activity which do not translate to the UK context [31]. Studies with combined mixed populations (BME and non-Black) involved extraction of BME population data only. This is response to scarcity of primary studies in this research field. Qualitative and mixed-method studies that reported barriers to engaging BME members in physical activity were included.

**Table 1:** Inclusion and exclusion criteria

Parameters	Inclusion criteria	Exclusion criteria
Location	Studies conducted in the UK	
Language	Studies written in English	
Population	Studies which included BME groups^a^ within ages 18–65 years.	Studies with non-BME groups
Outcome	Studies which reported findings of barriers^b^ to physical activity from the perspective of BME groups.	Studies with no reported analysis of barriers.
		Studies which reported findings of barriers to physical activity only from the perspectives of health providers, or Caucasians
		Studies with no quotes to support findings
Study Type	Primary studies which use qualitative methods to collect data and report their findings (mixed methods that included qualitative reports).	Studies with only quantitative or secondary analysis

^a^BME groups in the UK context; African, Caribbean, Indian, Pakistani, Bangladeshi and Nepalese. BME groups: Black and Minority Ethnic groups

^b^Separate analysis of barriers to physical activity among BME groups where studies included other population groups e.g. Caucasian)

### Critical appraisal

Duplicate, independent quality assessment was conducted using a modified version of the Critical Appraisal Skills Programme (CASP) [32] with a fourteen-item checklist as used by other meta-ethnographers [26–28].

### Analysis

**Fig. 2:**
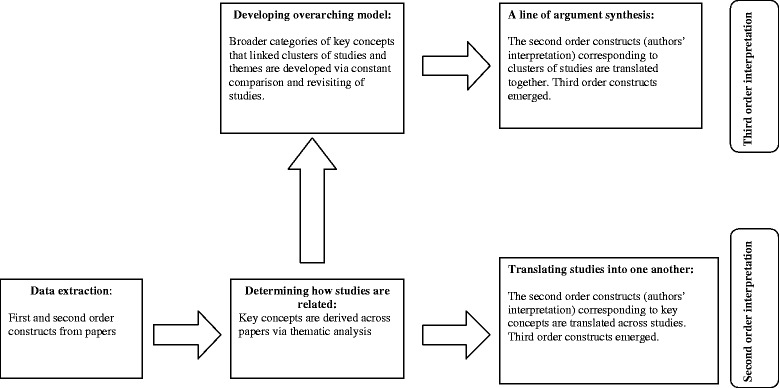
The Flow Diagram of Multi-staged Data Synthesis. A flow diagram shows the stages of meta-ethnography in qualitative synthesis. The stages are the processes of identification of key concepts, translation of studies into one another, and generation of ‘line of argument’

Data extraction and quality assessment were undertaken independently by two researchers (S.K and V.P-E). Figure 2 summarises the flow diagram of analysis. Meta-ethnography aims to achieve second-order interpretation and third-order interpretation of data. The process began with extraction of data called *first-order constructs* (i.e. the study participants’ interpretations of experience) and *second-order constructs* (i.e. the study authors’ interpretations of participants’ experience) from included studies. The third-order constructs are interpretations derived by systematic review team from first-order and second-order constructs.

For the second-order interpretation, the review team determined how studies were related by identifying similar findings across the studies (reciprocal analysis) [26] or identifying how studies refute each other’s findings (refutational analysis) [26]. This analysis was conducted in chronological order of included studies by three reviewers (S.K, R.J and M.W). The relationship between studies was demonstrated by the emergence of key concepts following re-organisation or juxtaposition of concepts across studies. The reviewers then translated the findings across the studies (by focusing on second order constructs) under each key concept. This completed the second-order interpretation by the review team.

The third-order interpretation resulted in generating overarching concepts identified by the wider review team (S.K, V.P-E, R.J and M.W) via constant comparison of the first, second and third-order constructs. The emerging overarching concepts across studies and their interpretation provided further insight and identification of theory and policy implications.

## Results

### Study characteristics

From the 2,986 references identified, 14 studies satisfied the inclusion criteria (Fig. 1). The studies were published between 1997 and 2011, and included 175 participants (18–65 years). The design and focus of the included studies varied. Three studies evaluated intervention programmes [33–35], and four explored knowledge and attitudes to lifestyle risk to developing diseases such as heart disease [36, 37], Type 2 Diabetes [38, 39]. The seven remaining studies explored physical activity experiences of participants that were unrelated to a specific clinical condition [30, 40–45].

Five studies were conducted in Scotland [30, 35, 38, 41, 43]; the remaining studies were conducted in England. All studies considered perspectives of BME groups, although two also included views from health professionals [39], as well as staff from Health Authorities and leisure centres [34]. Ethnicity was described by all studies, with 10 out of the 14 studies restricted to South Asians who were mainly Indian, Pakistanis, and Bangladeshi [30, 33–39, 44, 45]. The distinction between first generation migrants and UK born BME individuals were poorly reported in included studies. Four studies combined mixed populations of South Asians, majority populations and/or African-Caribbean [40–43].

Whilst some studies included participants aged over 65 years [37, 38, 42, 44], the mean age in each study was less than 65 years, hence their eligibility for inclusion in this review. Five of the studies expressed women’s views only and it is important to note that all of these involved South Asian individuals [34, 36, 37, 42, 43]. Table 2 summarises data from the included studies. Table 3 presents the results of the quality assessment performed on included studies.

**Table 2:** Study characteristics of papers that were synthesised (continued over two pages)

	Study Author Year of Publication (N = 14)	UK Location(s)	Qualitative methods	Participants’ characteristics	Recruitment	Aim(s)
1	Ahmad (2011) [45]	London	Participant observation & Semi-structured interviews	16 Muslim Women’s Football Team members (mostly South Asian heritage). Age 18–26 years.	Recruited via training centres	To explore the experiences and perceptions of the players in the British Muslim Women’s Football Team (BMWFT) are located within British football.
2	Carroll *et al.* (2002) [34]	Bradford, Leicester, East Lancashire and Birmingham.	In-depth interviews and focus groups	35 South Asian Muslim women (Pakistani and Bangladeshi)	Recruited via GP for individuals on the EoP schemes	To undertake case studies of exercise on prescription schemes in which provision is made for South Asian Muslim women in order to note good practice and any issues arising.
3	Farooqi *et al.* (2000) [36]	Leicester	Focus groups	44 South Asians, n = 20 females, n = 24 males. 11 Muslim, 22 Sikh, 11 Hindu. Mean age 53.5 years. Hindi, Gujarati, Punjabi	Recruited via letter from patients’ GP and opportunistic recruitment based on attendance to community centre.	To identify key issues relating to knowledge of and attitudes to lifestyle risk factors for CHD among South Asians aged over 40 years
4	Grace *et al.* (2008) [39]	London	Focus groups & semi-structured interviews	Bangladeshi people without diabetes. n = 37 males, n = 43 females. Bengali and Sylheti	Recruited via community centres, mosques, and GPs.	To understand lay beliefs and attitudes, religious teachings, and professional perceptions in relation to diabetes prevention in the Bangladeshi community.
5	Jepson et al. (2008) [30]	Aberdeen, Glasgow and Edinburgh	Focus groups	49 parents from Pakistani, Indian and Bangladeshi. Age 20-40 year.	Recruited via gatekeepers (local group staff/co-coordinators).	To explore the barriers, facilitators, motivators and types of activities among South Asian
6	Keval (2009) [44]	Midlands, North West and South East England	In-depth interviews	Type 2 Diabetes patients from South Asian (Hindu, Gujarati). Age 40-88 years. More than half under 65 years. N = 8 females, n = 10 males	Recruited through purposive and snowball sampling.	To explore experiences on management of type 2 diabetes among South Asian.
7	Lawton *et al.* (2006) [38]	Edinburgh	In-depth interviews	Diabetic patients of Indian and Pakistani origin. n = 15 males, n = 17 females. Age 30s -70s. Half were in their 40s and 50s	Recruited via letters from GPs.	To explore South Asian diabetic patients’ perceptions and experiences of undertaking physical activity as part of their diabetes care.
8	Netto *et al.* (2007) [35]	Edinburgh	Focus groups	55 people from India (mostly Sikh), Pakistan and Bangladesh (mostly Muslim).31 females, and 22 males. Age over 16 years.	Recruited verbally on attendance to clinic.	To explore how service user views and perspectives can be used to enhance the effectiveness of targeted CHD prevention initiatives
9	OPEN space (2006) [43]	Edinburgh	Focus groups	Women over 25 years of age. 5 BME members in 41 total participants. Jamaican, Bangladeshi and Indian.	Contacts and local facilitators known to OPEN space research centre.	To explore the views of people from disabled people, minority ethnic group and socially deprived areas
10	Rai and Finch (1997) [40]	London	Focus groups	175 India, Pakistan, Bangladesh, African and Caribbean. Age 18–50 years	Knocking on people’s door and approaching people in selected localities, then a letter of invitation.	To investigate attitudes towards, and barriers to physical activity among South Asian and black communities in England
11	Rishbeth (2004) [42]	Sheffield and Leicester	In-depth interviews	20 Indian sub-continent and Asian Africans from east Africa: Zimbabwe, Uganda, Kenya. Ages19-70 year.	Recruited via community centres	To explore the experience of people who have migrated from a different continent, climate and culture to live in Britain. How do people experience immediate and ongoing ‘culture shock’ with respect to the outdoor environment?
12	Sportscotland (2001) [41]	Edinburgh	In-depth interviews	40 Black African, Caribbean, Indian and Pakistani. A range of ages (40+ years).	Recruited via gatekeepers.	To provide sportscotland with an insightful and actionable strategy that will eliminate the current barriers to sports participation amongst people from ethnic minority backgrounds.
13	Sriskantharajah and Kai (2007) [37]	Nottingham	Semi-structured interviews	15 CHD and Type 2 DM patients from South Asians; Indian, Pakistani, Bangladeshi, East African Asian, Sri- Lanka. Hindu, Sikh and Muslim. Mean age 52 years. More than half were under 65 years.	Recruited via GP	To explore influences on, and attitudes towards, physical activity among South
						Asian women with CHD and diabetes to inform secondary prevention strategies
14	Williams and Sultan (1999) [33]	Trafford	Semi-structured interviews	15 Overweight or obese Asian women. Age 26–55 years.	Recruited via letter to previous attendees of a service developed by council.	The purpose of this qualitative evaluation was to conduct longer-term follow-up of the women who participated in the pilot group. Their views on the group and reasons for no longer attending.

Papers listed in alphabetical order of authors

*EoP* exercise on prescription*, CHD* congestive heart disease*, BME* black and minority ethnic*, DM;* diabetes mellitus*, GP* general practice

**Table 3:** Quality criteria and results

Study	Ahmad (2011) [45]	Carroll *et al.* (2002) [34]	Williams and Sultan (1999) [33]	Farooqi *et al.* (2000) [36]	Grace *et al.* (2008) [39]	Jepson *et al.* (2008) [30]	Keval (2009) [44]	Lawton *et al.* (2006) [38]	Netto *et al.* (2007) [35]	Rai and Finch (1997) [40]	Rishbeth (2004) [42]	OPENspace (2006) [43]	Sportscotland (2001) [41]	Sriskantharajah and Kai (2007) [37]
Is this study qualitative research?	+	+	+	+	+	+	+	+	+	+	+	+	+	+
Are the research questions clearly stated?	~	+	+	+	+	+	~	+	+	+	~	+	+	+
Is the qualitative approach clearly justified?	+	+	+	+	+	+	+	+	+	+	+	+	+	+
Is the approach appropriate for the research question?	+	+	+	+	+	+	+	+	+	+	+	+	+	+
Is the study context clearly described?	~	~	~	+	+	+	+	+	+	+	~	~	~	+
Is the role of the researcher clearly described?	~	~	~	~	~	~	-	~	~	~	~	~	~	~
Is there a connection to an existing body of knowledge or theory?	+	+	-	~	+	+	+	+	~	+	~	-	+	+
Is the sampling method clearly described?	~	+	+	+	+	+	~	+	+	+	~	+	~	+
Is the sampling strategy appropriate for the research question?	+	+	+	+	+	+	+	+	+	+	+	+	~	+
Is the method of data collection clearly described?	~	~	~	+	+	+	+	+	+	+	+	+	~	+
Is the data collection method appropriate to the research question?	+	+	+	+	+	+	~	+	+	+	~	+	+	+
Is the method of analysis clearly described?	~	+	~	~	+	+	~	~	~	+	-	-	~	~
Is the analysis appropriate for the research question?	~	+	~	~	~	+	~	+	+	+	~	~	+	~
Are the claims made supported by sufficient evidence?	+	+	~	+	+	+	~	+	~	+	~	~	+	+

+ = ‘Yes’, ~ = ‘Unclear’, − = ‘No’. Fourteen-item Checklist on Modified version of CASP tool. Source: Atkins *et al.* BMC Medical Research Methodology 2008, 8

### Main themes that emerged from the data synthesis

Four themes emerged from the findings of included studies (i.e. from first and second- orders). Each theme is described separately.

#### Perception

There was mixed perception of physical activity either as a formal separate activity or as part of everyday lives [30, 34, 36–42] (Additional file 1: Table S1). Some South Asian groups perceived physical activity as inappropriate or unnecessary or adding no value; to them, physical activity was perceived to cause harmful effects [41]. For example, physical activity was perceived harmful as a cause more weakness or disease [35], and as a reflection of selfish activity to abandon other responsibilities [37]. Furthermore, physical activity was perceived among Bangladeshi people as harmful and cause social sanction of gossip and laughter among women [39]. Physical activity was perceived to be absent from their culture. For example, among the South Asian groups, physical activity was perceived as “Western” culture which was external to their own lifestyle and BME individuals were not able to incorporate it easily into their lives. The requirements of special clothing or undertaking activities at designated places such as gymnasiums reflected the notion that physical activity was perceived as formal and separated activity rather than BME cultural activities [39]. This perception of an absence of exercise culture originated from the participants’ country of origin where there was limited childhood exposure or experience of activities perceived as “Western culture”. Cultural restrictions, lack of role models in physical activity or sport from BME communities, and the poorly promoted healthy lifestyles were described to explain their limited exposure in their country of origin [30, 37, 40, 41].

Within the included studies, perception of disease causation or risks and health beliefs were revealed among individuals from South Asian groups who held strong health beliefs and perceived that physical activity had no preventive role in diseases [30, 33, 35, 38–40] (Additional file 1: Table S1). This perception was shared by a variety of individuals with or without co-morbidities such as diabetes and obesity. For individuals with an Islamic background, ageing and external locus of control (e.g. God) were considered causes of disease with little or no control being derived from human activities (including physical activity) to prevent them [35, 38]. Therefore, some BME individuals would not engage in physical activity either as a preventive measure or as treatment for specific health conditions.

The perceived fear of racial or religious discrimination among some BME individuals was a barrier to engaging in physical activity in the UK [30, 34, 35, 39, 41, 45] (Additional file 1: Table S1). For example, some Muslim women, especially from South Asian groups, reported avoiding facilities which were unfamiliar to them or where they might feel unsafe because of fear of either personal or institutional racial discrimination from either the Caucasian population or members of the same BME group [45]. Perceived personal discrimination from members of the same BME group could occur when their participation in sport was condemned by their own BME community. For example, females participating in football [45]. Some female participants perceived discrimination from Caucasian groups at public facilities when traditional clothes rather than formal sportswear were worn [30]. Closely related to this was the fear of crime and physical attack perceived by many individuals across BME groups and deterring them from outdoor physical activities. The fear of crime and physical attack was closely related to perceived fear of racial discrimination living in disadvantaged areas*.*

#### Cultural expectations

Within the identified studies, cultural and religious norms were identified as deterrents to engaging in physical activity [30, 33–35, 38, 41, 45] (Additional file 2: Table S2). These norms included: maintenance of Islamic or South Asian dress codes; curtailing movement of women outside the home and female cultural obligations after marriage. This suggests that the desire to observe these norms by some South Asian women or Muslim women being stronger than the desire to be physically active. More so, there was a fear of breaking the rules or acting outside these norms to avoid condemnation from members of same BME group. The threat of the disappearance of traditional cultural values was also another reason BME members would desire to observe these norms. Therefore, individuals from BME groups (especially South Asian women or Muslim women) found it difficult to meet expectations of their traditions as well as becoming sport individuals [45]. The lack of culturally-sensitive indoor facilities and services deterred some BME individuals from engaging in physical activity [30, 34, 38–41].

Cultural expectations in some BME groups are religious and culturally based. Sometimes, South Asian individuals expect physical activity facilities to promote or incorporate their religious and cultural practices, for example, single-sex facilities and same-sex instructors or life-guards [38, 41]. These cultural expectations were embedded in their religious beliefs of segregated environment for both genders (as also observed during Muslim prayers). The gender identity of South Asian women was pronounced as dictated by cultural norms and family obligations. Emphasis was placed upon South Asian women to stay indoors, attending to domestic chores, and prioritise family responsibilities over their independence and freedom [37–39, 41]. In this way, to the community groups, modesty as expected by religious beliefs was preserved by both genders in Muslim or South Asian communities. Whilst culturally-sensitive facilities exist, there was a, lack of awareness among BME groups of their existence [30, 41].

The time constraints produced by competing cultural priorities limited participation of some BME individuals (both South Asian and African origin) in physical activity [30, 38, 40, 41]. This tended to affect South Asian women more than their male counterparts because of heavier cultural responsibilities or expectations after marriage [38]. Some BME individuals were unable to understand information or share information about their needs due to language barriers [34, 36, 39, 41]. This problem was more pronounced among some older South Asian groups and first generation migrants. Therefore, there were limitations to healthy lifestyle choices (including physical activity) or decisions that could be made by BME individuals.

#### Personal barriers

Time constraints due to social and work commitments limited participation in physical activity among some BME individuals [30, 34, 35, 37–41] (Additional file 3: Table S3). Greater emphasis was placed on work commitments (e.g. long working hours) over physical activity for financial stability in the UK especially following migration [30–41]. For female South-Asian participants, there was pride and priority of family commitment (childcare and household management) over physical activity. Our interpretation suggests low priority in having control over personal health and social freedom in these groups.

The influence of health problems on South Asian groups’ participation in physical activity was reinforced by their health beliefs which focused on the harmful effects of physical activity rather than its benefits [33, 37, 38, 40]. For example, the belief that excessive sweating and increased heart rate associated with physical activity was perceived as illness rather than normal by-products of exercise [38]. Therefore, the fear of provoking physical symptoms rather than reported ill-health was a pronounced barrier among some BME individuals in engaging in physical activity.

Within the studies, lack of confidence and motivation was common to all BME groups as personal barriers to physical activity [30, 33, 35, 40]. There was no perceived enjoyment or motivation to participate in physical activity because it was perceived as a formal and separate activity from BME everyday lifestyles. The BME individuals exhibited lack of confidence which was compounded by communication barriers, an alien environment and the lack of social networks for carrying out physical activity [38, 41, 42]. First generation migrants in particular lacked confidence and faced challenges due to limited social networks, or lack experience of new services or skills needed for physical activity that were not familiar to them. As such, initiating the use of neighbourhood services was perceived as being difficult by BME individuals.

#### Factors limiting access to facilities

Various external factors were identified that limited participation by some BME individuals in physical activity in the UK. These included: climate [30, 38, 39, 41, 44], distance to sports facilities [30, 33, 37, 40, 41], lack of information [34, 35, 37, 40, 41], cost [34, 39–41], lack of childcare facilities [30, 34, 39, 40], and accessing facilities in unfamiliar neighbourhoods [30, 38, 42] (Additional file 4: Table S4). Many of these factors were inter-dependent. For example, access to distant facilities was constrained by lack of transport, time constraints and the unfamiliar environment. Individuals also reported that lack of familiarity about their physical environments exacerbated the feeling of being unsafe and vulnerability among some female respondents from South Asian and African groups; thus increasing the difficulty of seeking out information about physical activity. Most of these factors or barriers under the category of ‘factors limiting access to facilities’ were not specific to BME individuals. Although the cost of exercise may be more problematic for some BME individuals (compared to majority population), expenditure on exercise by some BME individuals was considered wasteful and of low priority [38]. This may be a reflection of socio-economic issue as people from most minority ethnic groups are generally more deprived in terms of socio-economic status [46]. This behaviour among some BME members might also have been shaped by their experience of some BME individuals using facilities free-of-charge in their country of origin, and then contributing to the perception of physical activity being expensive in the UK.

### ‘Line of argument’ synthesis

The synthesis provided in this section attempts to construct the interpretation of over-arching concepts identified from the 14 included studies. The emergent concepts provide insight into future potential interventions. The overarching concepts derived in this synthesis indicated that the barriers which influence physical activity behaviour among BME groups exist at individual, physical environment and organisational levels. This relates to the socio-ecological framework that proposes many of the determinants of health are understood as influences within and on individuals by social groups, environments and larger society of which the individual is a part [35]. Table 4 presents the summary of information on the ‘line of argument’ synthesis.

**Table 4:** Line of argument synthesis

Levels; synthetic headings	Third order constructs (themes)	Third order interpretations (Reviewers’ interpretations)
Individual	First generation migrants versus later generation migrants	The degree of socio-cultural barriers to physical activity exhibited by first generation is greater than in the later generations.
		First generation migrants exhibit weaker interpersonal relationships that result in poor social network and differential style in negotiating the health system and facilities for physical activities.
Community	Unfamiliar environments versus familiar environments	There is greater attention and emphasis placed on carrying out physical activity in gymnasium than in familiar places like school, work, and religious centres.
	Barriers similar to general population versus barriers specific to BME groups	The majority of barriers emerging from physical environments are similar to those identified in general population. E.g. distance, finance, bad weather.
Organisational	Lack of inclusive services and research for all people as influenced by organisational structure and practices.	Most organisations and policy-makers do not consider potential risk of ‘institutional racism’ in their practices, this limit participation of BME groups e.g. non-inclusive single-sex facilities, lack of specific information to help BME groups, and lack of training of staff.
		There are existing culturally competent facilities but poor marketing of existing services affects awareness of services that are culturally competent. Service providers not recognising that they may need to offer different services or use different settings to promote physical activity (e.g. in the community, workplace or religious settings)
		A limitation of current research in recognising cultural activities that are physical activities might have led to health promoters not being adequately informed on how to address barriers among BME groups.

#### Individual level barriers

Clusters of studies reported that barriers to physical activity occur at an individual level and are influenced by BME groups’ socio-cultural backgrounds and interpersonal relationships [34, 36–42]. Following migration into the UK, ‘self identity’ played a role in the attitude of an individual towards physical activity. The attitudes of some BME individuals towards physical activity were shaped by the collective cultural beliefs and perceptions which individuals held before migration to the UK [39–41]. However, this influence from socio-cultural background on individuals would be expected by researchers to attenuate over a period of time [38]. This synthesis suggests that the degree of socio-cultural barriers to physical activity exhibited by first generation BME individuals was more pronounced than later generations. With regard to the influence of socio-cultural background, as a result of poor interpersonal relationships experienced by BME individuals in the UK and difficulties in communication with health professionals, BME individuals may be disempowered in participation of physical activity.

**Fig. 3:**
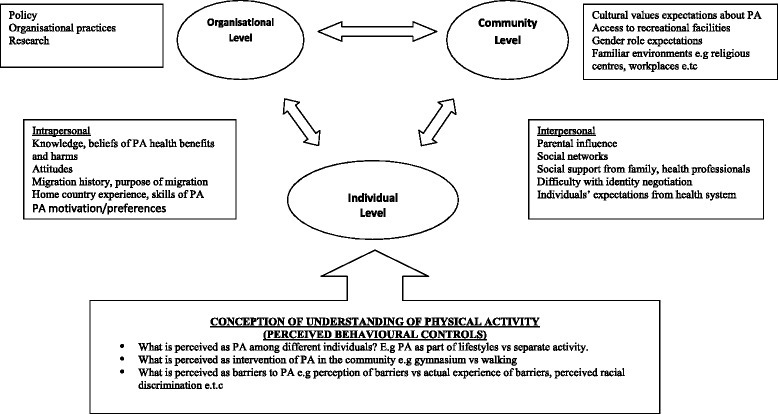
Influences on Physical Activity among BME groups, A Conceptual Model. The model describes influences at individual, community and organisational levels on behaviour towards physical activity among BME groups. The inclusion of social concept ‘conception of understanding of physical activity’ into socio-ecological model and its influence on individual behaviour

As BME individuals settle into UK communities, their interpersonal relationships may influence participation in physical activity. Six studies indicated how interpersonal relationships were affected by language barriers, lack of confidence and poor social network [34, 36, 37, 39, 41, 42]. Among some BME individuals, there was a problem with self-identity as well as communicating individual needs in terms of health information and physical activity facilities. However, the concepts of physical activity among BME individuals played a major role. The varied concepts of physical activity BME individuals were shaped by cultural factors, socio-economic background, knowledge and past experience. In view of these differences, a conceptual model (Fig. 3) was constructed that demonstrates the influence of conceptual understanding of physical activity among individuals, not merely ‘practical’ understanding of physical activity barriers. This is a key finding of this study which described the construct when incorporated into socio-ecological model may be relevant to developing theory or interventions related to physical activity behaviour among BME individuals.

#### Community level barriers

Many barriers to physical activity existed at a community level and were clustered as either ‘unfamiliar environment’ versus ‘familiar environments’ or as ‘barriers similar to general population versus barriers specific to BME groups’.

In all the included studies, BME groups focused on the attendance at facilities e.g. gymnasiums, leisure centres, to engage in physical activity. There was little focus on participation in physical activity in familiar environments in which BME groups were likely to carry out their daily activities, for example, school, work and their neighbourhood. The synthesis suggests that facilities which are outside familiar environments of BME individuals reinforced the barriers which limit access to facilities. The synthesis reinforced how BME groups have different perceptions of interventions in the environment by focusing on carrying out physical activity at mainly recreational centres such as gymnasiums. This reflects the misperception of physical activity by many individuals from BME groups as a formal and separated activity rather than recognition of certain lifestyle activities in their culture. More so, this may translate to a lack of awareness of interventions at these familiar places such as school, religious centres and workplace. Some barriers were specific to only BME groups whilst others are similar to those experienced by the general population [30, 34, 36–38, 40]. For example, distance, finance, bad weather, and time constraints due to competing responsibilities were also experienced by the general population. The predominance of activities related to religious or cultural practices were found among South Asian populations. In this study, these were barriers at community level that were specific to a BME group and might not necessarily be found in other BME groups.

#### Organisational level barriers

Clustered within seven studies were descriptions of limitations of strategies used by organisations to promote physical activity among BME groups [30, 34, 35, 38–41]. The authors of these studies perceived that BME individuals were disadvantaged by problems in organisational structures including inadequate advertising of existing services and failure to tackle institutional racism. The failure to also embark on research to help engage BME groups in physical activity existed at organisational level. In relation to poor marketing of existing services, although there were existing services, the use of inappropriate channels of communication or poor partnership with organisations among BME communities influenced the awareness of BME groups of initiatives that facilitated physical activity [35, 40].

With regard to institutional racism, the non-inclusiveness of services prevented participation of BME groups in physical activity [39, 41]. Many service providers did not recognise that they might need to offer different services or use different settings to promote physical activity (e.g. in the community, workplace or religious settings). The absence of services such as single-sex facilities and the reluctance of organisations or policy-makers to provide specific information to help BME groups contributed to institutionalised racism. In another dimension of barriers at organisational level, BME members and health promoters had not benefited from research to guide decision making on cultural lifestyles. Health promoters and professionals might have been inadequately informed on how to address barriers to physical activity among BME groups. Overall, BME individuals had no immediate control over the barriers experienced at organisational level.

## Discussion

### Principal findings

The health inequalities related to physical inactivity are recognised when comparing BME groups with the people from European origin [6, 7]. However, there is weak evidence of interventions that support efficacy in improving the behaviour of physical activity and overcoming barriers to physical activity among BME groups [47]. This study provides insight into previously under-acknowledged factors contributing to low physical activity levels among BME groups. Hence, the review did not focus on low physical activity levels, but rather wanted to know the general barriers to physical activity that were identified by BME populations. It is acknowledged that these barriers might be different to people with low or high physical activity levels. Of particular importance is the finding that barriers to physical activity among BME groups exist at individual, community and organisational levels. Significant to this field of research, this study identified the social construct of ‘individual perception and understanding of physical activity’ as being key to theoretical models and intervention.

The findings reflect the socio-ecological model of determinants of physical activity described at individual, community and organisational levels [48, 49]. At each level of the socio-ecological model the barriers varied with individual perceptions, cultural expectations, personal reasoning and factors limiting access to facilities. Whilst there are demonstrable differences in health status between BME groups and the majority groups, the causes are related to experience with different levels of resources, different levels of exposure to health hazards and life-course effects including migration, and people may experience socio-economical differences in health. Irrespective of upward social mobility or changes in health behaviour, an ethnic group may maintain a common belief, culture, meanings and interpretation from one generation to another. This may have significance in levels of reported poor general health and physical activity levels observed between generations [50]. Furthermore, the effects of migration from rural to urban (for example, UK) setting could explain the attitudes of some BME individuals towards physical activity as shaped by the collective cultural beliefs and negative perceptions which individuals held before migration to the UK [39–41]. However, researchers argued that migrants in rural areas already acquired physical activity levels similar to urban regions [51]. There is need for future research to explore patterns of physical activity among migrants from rural settings that may be applicable to UK. Efforts to identify and promote range of behaviour (including leisure or non-leisure, occupational or household tasks) among migrants should be integral to strategies to promote physical activity [52].

Findings from this meta-ethnography are consistent with reviews that focused mainly on elderly South Asian elderly populations [47, 53]. The studies have shown that disempowering effects of socio-cultural background and poor interpersonal relationships on BME individuals’ identity are responsible for difficulty in self confidence, self-effectiveness and self-esteem in participating in health programmes. Consequently, some BME individuals are faced with challenges of identifying their needs, expressing themselves, sharing experiences and using personal and collective resources (including communication with health professionals) to overcome health and physical activity related problems. Our synthesis suggests that the effect of social-cultural background is attenuated in later generations, although individuals from BME communities may perceive the act of conforming to Western culture of seeking information about physical activity to constitute identity threat [54]. This implies that behavioural and social approaches should aim to empower BME individuals who may feel socially excluded following migration in the UK [55]. The sense of connectedness in group based interventions in the community may establish social networks, motivation and psychological supports that increase the likelihood of maintaining and improving levels of physical activity [55]. There is evidence that changes to policies and environmental characteristics promote active transport, increase safety, improve affordability and facilitate access to physical activity [47, 55]. Therefore, working in partnership with BME groups during policy implementation may reflect the interest of BME groups and further empower minority ethnic communities.

This meta-ethnography provides insight into understanding physical activity and future potential interventions in addressing low participation among BME individuals. For example, differences in perceptions used by BME individuals to describe physical activity as a formal and separate activity rather than recognition of certain lifestyle activities in their culture, and the focus on recreational centres such as gymnasiums as the main intervention site in the environment. Therefore, there are missed opportunities to engage BME groups in their familiar physical and social environments such as settings and communities in which BME groups live, work and worship. This reinforces that social and built environments are determinants of physical activity behaviour [49]. The practical implication is that intervention strategies need to explore opportunities to address the mismatch in perceptions of physical activity, health beliefs and expectations from those of health professionals. In recognition of individual differences that exist within each group, this implies that an intervention may not be effective for all in the group; hence, an individual approach to health promotion is still relevant for providing culturally sensitive programmes. This approach may help overcome the conflict between cultural identity and the notion of adopting Western culture. The leaders of physical activity may want to be culture and gender specific using role models from same BME communities to alleviate the threat of their own cultural identity. For example, a group activity for South Asian females may not be effective if led by Caucasian man. Gender-specific interventions for BME female could include flexible dress code and delivery of interventions at religious or community centres.

Theoretically, this study contributes a more nuanced understanding of the concept of physical activity. The identification of the social construct of ‘individual perception and understanding of physical activity’ that is relevant to theoretical models and interventions. Because many of the influences were perceived rather than being actual experiences, this social construct can be linked to ‘perceived behavioural beliefs or controls’ used by behavioural theory – Theory of Planned and Reasoned Action to help explain barriers to physical activity [56]. Understanding the concept of physical activity is also useful to explain influences on participation in physical activity or access to resources on individuals. A recent review of barriers to engaging in physical activity among BME individuals in the UK, presented a narrative synthesis of results, not focused on developing new interpretation in the form of a plausible hypothesis, conceptual framework or policy [57]. However, this study goes beyond individual studies and thus contributes to conceptual and theoretical development. To reduce social inequalities between BME groups and the majority population, it is difficult to develop specific intervention for any given BME group. However, the varied individual perception and understanding of physical activity is a significant ‘perceived behavioural control’ or social construct to be considered in interventions adapting ecological model [48] (Fig. 3). Interventions need to focus on the task of overcoming the perceived behavioural controls among BME individuals at individual, community and organisational levels.

### Strengths and limitations

A strength of this study is the rigour with which the review and synthesis was conducted comprising the robust systematic search that included experts’ resources, grey literature, and the focus on UK-only studies. The conceptualisation of the synthesis to individual, community and organisational levels has implications for practice and policy. This synthesis identified new insights and provided fuller understanding of the experiences of BME individuals related to barriers to engaging in physical activity. The synthesis through meta-ethnography goes beyond identification of practical barriers by revealing the complex interplay of variables that contribute to barriers including individual migration history, interpersonal relationships, and the ways in which policy and physical environments act as external influence on BME groups in physical activity participation.

Furthermore, the use of quality assessment in this review was part of the selection criteria and also reflected robustness of resulting synthesis [58]. Whilst some of included studies were inadequately reported, they contributed unique dimensions within themes. For example, Rishbeth [40, 42] added to the theme of ‘lack of confidence’ regarding the disconnection with the physical environment due to migration changes which diminishes BME groups’ confidence and skills to explore physical activity in the UK. This was a unique dimension that reinforced explanations of lack of confidence due to communication barriers and lack of social support when carrying out physical activity. Although views on inclusion of poorly reported or low quality papers into qualitative synthesis are inconsistent, calls for consolidated standards of reporting qualitative synthesis may address the situation in the future [58].

With regard to study limitations, the potential to obtain substantial information on barriers to physical activity may have been limited by inclusion of primary studies that targeted more than one lifestyle behaviour. For example, unhealthy diet was another behaviour focused upon in four studies [33, 35, 36, 44]. However, in practice, unhealthy behaviours such as unhealthy diet, excessive alcohol consumption and physical inactivity are often clustered [59] and the knowledge of health promotion across the clustered behaviour adds to development and evaluation of interventions that target multiple health behaviours to achieve population gains [59].

As most of the included studies targeted the South Asian population, the synthesis of findings (especially perceptions and cultural barriers) may not be applicable to other main ethnic groups in UK context including African-Caribbean populations. The study acknowledges the limitation of adopting a holistic approach of including a wide age group (18–65 years) and all ethnic groups. However, this approach provided an opportunity to review what is known about a range of minority groups. This study re-iterates the paucity of studies in this field across ethnic groups and lack of sub-analysis of individual ethnic groups in the UK. Future research may need to consider sub-analysis including age, years of migration, individual ethnic groups and distinction between first generation migrants and UK-born BME individuals.

## Conclusion

The study was conducted to address the gap in evidence on effectiveness of health promotion interventions in BME groups and observed increasing health inequalities between BME groups and general population attributed to differences in risk behaviours like physical inactivity. The study has identified the mechanisms that influence BME individuals’ participation in physical activity. In particular, the socio-ecological model that explains barriers to physical activity has implications for practice, policy and further research. Interventions to enhance physical activity among BME groups need to acknowledge the barriers to physical activity arising from individual’s perception of physical activity. Key to developing successful interventions is the need for adopting several empowerment approaches at individual, community and organisational levels. Such approach, incorporating findings of this study (undertaken in a UK context) could contribute to reducing social and health inequalities arising from differences in physical activity levels between BME groups and general population.

## Additional files

Additional files 1: Table S1. Perception; key themes, second constructs and translations of one study into another. This table displays similar and opposite themes under the concept of ‘perception’ from across studies. The themes were translated into one another to produce second-order interpretation. This is a multipage table to be viewed as hyperlink. File exists in .txt format. Additional files 2: Table S2. Cultural Expectations; key themes, second constructs and translations of one study into another. This table displays similar and opposite themes under the concept of ‘cultural expectations’ from across studies. The themes were translated into one another to produce second-order interpretation. This is a multipage table to be viewed as hyperlink. File exists in .txt format. Additional files 3: Table S3. Personal Barriers; key themes, second constructs and translations of one study into another. This table displays similar and opposite themes under the concept of ‘personal barriers” from across studies. The themes were translated into one another to produce second-order interpretation. This is a multipage table to be viewed as hyperlink. File exists in .txt format. Additional files 4: Table S4. Factors limiting access; key themes, second constructs and translations of one study into another. This table displays similar and opposite themes under the concept of ‘factors limiting access” from across studies. The themes were translated into one another to produce second-order interpretation. This is a multipage table to be viewed as hyperlink. File exists in .txt format.
