# Supplementary material for: Understanding the complex interplay of barriers to physical activity amongst black and minority ethnic groups in the United Kingdom: a qualitative synthesis using meta-ethnography
Source: BMC Public Health. 2015 Jul 12;15:643. doi: 10.1186/s12889-015-1893-0 (PMC4499183; doi:10.1186/s12889-015-1893-0)
Supplement: Additional files 1: Table S1. — Perception; key themes, second constructs and translations of one study into another. This table displays similar and opposite themes under the concept of ‘perception’ from across studies. The themes were translated into one another to produce second-order interpretation. This is a multipage table to be viewed as hyperlink. File exists in .txt format. [file 12889_2015_1893_MOESM1_ESM.doc]

**Supplemental Table S1:** Perception; key themes, second constructs and translations of one study into another (Continued over 4 pages)

| **Perception; key themes** | **Extracted second order constructs**  **(authors’ own words or paraphrase)** | **Summary of translation across studies (Second order interpretation)** |
| --- | --- | --- |
| **Perception of ‘meaning and interpretation of exercise’** | Exercise regarded as formal vigorous activity not as a lifestyle issue’. **Farooqi *et al.* (2000)**  Sporting exercise for women ...was seen as inappropriate and liable to meet with the social sanction of gossip and laughter’. **Grace *et al* (2008)**  ‘Particular activities which they did not feel were relevant to them or their communities. e.g. hill walking, rock-climbing) differed’ **Jepson *et al.* (2008)**  ‘Certain physical activities during their everyday lives are not necessarily recognized as such by themselves nor by researchers and/or health-care professionals’ **Lawton *et al.* (2006)**  BME members are unclear about the meaning of physical activity in terms of frequency, duration intensity… had low priority to fit into leisure time as it is not seen to be enjoyable and relaxing, rather it causes physical exhaustion. Physically inactive BME members do not consider physical activity during work, or travel to and from work as physical activity …There is no value in doing further physical activity if you are fit already’ **Rai and Finch (1997)**  ‘Within this context, sport was seen to be an unnecessary or inappropriate aspect of a woman's lifestyle. As such, they had faced considerable pressures to stop doing any sport’ **Sportscotland (2001)**  ‘The notion of ‘exercise’ that goes beyond daily work was perceived by some as a selﬁsh activity’ **Sriskantharajah and Kai (2007)** | Mixed conception of physical activity as either a formal separate activity or as part of everyday lives among BME individuals. Some individuals from BME groups fail to recognise some of their lifestyles as physical activity. |

**Supplemental Table S1 (Continued):** Perception; key themes, second constructs and translations of one study into another

| **Perception; key themes** | **Extracted second order constructs**  **(authors’ own words or paraphrase)** | **Summary of translation across studies (Second order interpretation)** |
| --- | --- | --- |
| **Perceived absence of exercise culture** | ‘Communities do not have a culture of exercise in the traditional Western sense of the word’. **Carroll et al. (2002)**  ‘Black and minority ethnic groups did not have the same cultural habits when visiting or using local woodlands or green space’. **Jepson *et al.* (2008)**  ‘Physical activity as a leisure pursuit was simply not a part of their cultural repertoire’. This was because of the cultural background and attitudes of their parents or older members of the community’. **Jepson *et al.* (2008)**  ‘Exercise in the Western sense (designated activities with special clothing, undertaken in special places such as gymnasiums) was seen as alien to the culture and identity of many first generation’ **Grace *et al* (2008)**  **‘**Their movements outside the home also appear to have been curtailed by social rules and cultural considerations, including the absence of a culture of exercise. These women found it particularly difficult to increase their physical activity as they had not been socialized into spending time outdoors, let alone to participating in sports’  **Lawton *et al.* (2006)**  ‘There is no physical activity at country of origin because it was a western concept. **Rai and Finch (1997)**  ‘Lack of a tradition in their culture of ‘Western’ exercise such as sport, noting that no one among their relatives did any form of such exercise’. **Sriskantharajah and Kai (2007)**  ‘Significant others themselves did not consider sport to be an appropriate behaviour – either due to cultural or religious considerations or a perceived lack of value in participating in sport. 'significant others'; these people have an important role to play in influencing the attitudes and behaviour of those close to them. **Sportscotland (2001)**  ‘Activities like going to woodlands, walking, or camping are most unlikely to be part of eastern cultures’ **OPEN space (2006).** | Physical activity perceived external (absent) to BME lifestyles, and related to ‘Western culture’. The lack of childhood exposure and experience of “Western” activities in the country of origin enhanced this perception and was reinforced by cultural restrictions, lack of role model and health promotion. |

**Supplemental Table 1 (Continued):** Perception; key themes, second constructs and translations of one study into another

| **Perception; key themes** | **Extracted second order constructs**  **(authors’ own words or paraphrase)** | **Summary of translation across studies (Second order interpretation)** |
| --- | --- | --- |
| **Perception of disease causation or risks and health beliefs** | ‘Perceived causes of diabetes included heredity and stress’ **Grace *et al.* (2008)**  ‘Diabetes weakened their bodies in ways which could not be reversed or delayed’. ‘Perception that ill-health is an inevitable and hence biographically anticipated feature of ageing’ ‘Blame external factors such as the will of Allah/God, genetic fatalistic notions seemed to have been reinforced by their belief that health, illness and death are pre-ordained by Allah/God’ **Lawton *et al.* (2006)**  ‘The most important predictors of outcome and compliance are patients’ beliefs about the cause and course of their disease’. ‘Perception of …external factors …they have little control,… belief that a combination of advancing age and doing more exercise would result in feelings of weakness’. **Netto *et al.* (2007)**  ‘Perception that they cannot lose weight without exercise machine **(William and Sultan 1999)**  ‘Physical activity only being important once a person had a specific medical condition, or were overweight, rather than it was being about preventing these conditions’.  **Jepson *et al.* (2008)**  ‘Belief that after certain age, physical activity could no longer provide benefits to the body. Opportunities for physical activity diminish with age and stages in life when there would be more responsibilities. **Rai and Finch (1997)** | Health beliefs that ageing and external locus of control (e.g. God) are causes of diseases with no influence from human activities (including physical activity) to prevent diseases. Physical activity not considered as modifying or preventive measures for diseases. |
| **Perceived fear of racial discrimination** | ‘Discrimination that Muslim women face from ‘within’ their groups as well as from the ‘outside’’ **Ahmad (2011)**  Concern shown by members of families and communities.…. Muslims suffer the added dimension of religious discrimination’. **Carroll *et al.* (2002)**  ‘Various experiences of racism (both institutionalised and personal). ‘Traditional’ South Asian clothing, sometimes became targets for racist remarks when out in public (personal racism) and sometimes experienced the institutional racism embedded in some services: the inability to access services because they were not truly women-only, or being prevented from entering a swimming pool wearing attire’ **Grace *et al.* (2008)**  ‘Feelings of …racial hostility’. **Netto *et al.* (2007)** | No interest to seek out facilities due to fear arising from personal and institutional racial or religious discrimination. There is also religious and gender discrimination from individuals within the same BME groups, outside the group (e.g. Caucasian) or organisational culture. |

**Supplemental Table 1 (Continued):** Perception; key themes, second constructs and translations of one study into another

| **Perception; key themes** | **Extracted second order constructs**  **(authors’ own words or paraphrase)** | **Summary of translation across studies (Second order interpretation)** |
| --- | --- | --- |
| **Perceived fear of racial discrimination**  **(Continued)** | Racial discrimination is not just about physical or verbal abuse but also includes institutional racism, by far the largest barrier. In terms of providing sport to this target group. … They may have come across a general reluctance from information-providers to give specific help or information, often felt to be due to a lack of knowledge on their part or inherent racism. **Sportscotland (2001)**  Personal safety in disadvantaged areas is also an issue for the majority population, but it would appear that racism sometimes compounds the problem of personal safety in these areas **Jepson *et al.* (2008)** |  |
| **Perceived Personal Safety** | ‘Issues of personal safety deter people from public spaces. Fear of being attacked is related to racial nature’ **Rai and Finch (1997)**  ‘Ethnic minorities considered themselves more at risk than others walking alone, and the countryside is seen as a more racist environment’ **OPEN space (2006)**  ‘Fear of crime,…. may explain the much lower levels of participation in formal exercise programmes in Muslim communities compared with Majority population populations, particularly for women. **Grace *et al.* (2008)**  Concern shown by members of families and communities. This may relate to safety issues, prevent South Asian Muslim women from attending some centres…. **Carroll *et al.* (2002)**  Personal safety in disadvantaged areas is also an issue for the majority population, but it would appear that racism sometimes compounds the problem of personal safety in these areas **Jepson *et al.* (2008)** | The fear of crime or physical attack limits outdoor activities. May be compounded by perceived fear of racial discrimination. |
