# Supplementary material for: Understanding the complex interplay of barriers to physical activity amongst black and minority ethnic groups in the United Kingdom: a qualitative synthesis using meta-ethnography
Source: BMC Public Health. 2015 Jul 12;15:643. doi: 10.1186/s12889-015-1893-0 (PMC4499183; doi:10.1186/s12889-015-1893-0)
Supplement: Additional files 2: Table S2. — Cultural Expectations; key themes, second constructs and translations of one study into another. This table displays similar and opposite themes under the concept of ‘cultural expectations’ from across studies. The themes were translated into one another to produce second-order interpretation. This is a multipage table to be viewed as hyperlink. File exists in .txt format. [file 12889_2015_1893_MOESM2_ESM.doc]

**Supplemental Table S2:** Cultural Expectations; key themes, second constructs and translations of one study into another.

| **Cultural Expectations; key themes** | **Extracted second order constructs**  **(authors’ own words or paraphrase)** | **Summary of translation across studies (Second order interpretation)** |
| --- | --- | --- |
| **Cultural or religious norms** | ‘hijab was not usually ‘tolerated’, and often hijab wearers were alienated in sport, … visible barrier to sport in Britain’. ‘Issues of identity….their struggle to compete in sport whilst observing the hijab illustrates the importance of religious belief, indicating that the hijab was something highly significant to each individual which could not be compromised in order to compete in football.’ ‘Consequences of breaking perceived rules of cultural adherence. .. experience verbal abuse from male Muslims; taunted for being ‘boyish’ and unfeminine **Ahmad (2011)**  ‘The main religious issue with regard to the male–female dynamic is the separation of the sexes and the maintenance of Islamic dress codes.’ **Carroll *et al.* (2002)**  ‘Their movements outside the home also appear to have been curtailed by social rules and cultural considerations. **‘**That some of the social and cultural factors described above will attenuate over time, especially amongst the later generations’. **Lawton *et al.* (2006)**  ‘Female participants in particular felt that they had (in some cases) been actively discouraged from going out and exercising outside the home. **Jepson *et al.* (2008)**  ‘adherence to social rituals and community norms around hospitality… threats to traditional cultural values, **Netto *et al.* (2007)**  ‘Some Muslim women, it was felt that their religion stipulates that women are not to undertake sport in any form whatsoever. This can be related both to the undertaking of sport per se (seen as appropriate for males only) as well as to what the activity may entail (such as wearing ‘inappropriate’ clothing). Within the Indian community, this was an issue for married women only. ‘**Sportscotland (2001)**  ‘Social factor is an important influence, in particular was been unable to go out of the house’. **William and Sultan (1999)** | Desire to observe a cultural and religious norm is stronger than desire to be physically active. It has a generational impact. It is also reinforced by fear of condemnation from inside members or threats of disappearance of traditional cultural values when norms not observed. |

**Supplemental Table S2 (Continued): Cultural Expectations; key themes, second constructs and translations of one study into another.**

| **Cultural Expectations; key themes** | **Extracted second order constructs**  **(authors’ own words or paraphrase)** | **Summary of translation across studies (Second order interpretation)** |
| --- | --- | --- |
| **Lack of culturally sensitive facilities** | ‘It is of vital importance that men do not supervise these women-only sessions. As with women generally, many Muslim women are also put off by communal shower areas and would prefer separate shower and changing cubicles’ **Carroll *et al.* (2002)**  ‘Mixed sex exercise classes were considered inappropriate for both women and men… these might still fail to preserve modesty or meet privacy needs’. **Grace *et al.* (2008)**  Sports facilities were often not providing culturally competent services. Modesty was an important concern for women with regard to undertaking physical activity on western sport’s attire and it was sometimes linked in with their religious beliefs. **Jepson *et al.* (2008)**  ‘Culturally sensitive facilities, such as single-sex sessions at local swimming pools with same-sex instructors’ **Lawton *et al.* (2006)**  ‘Inappropriate facilities……, nature of the facilities or dress requirements, female (or male) only environment... male instructor or lifeguard present.’ Lack of ‘people like me’ such as other Asian people playing football together. Lack of awareness of the existence of types of sport that people were either familiar with, or felt comfortable participating in.  **Sportscotland (2001)**  ‘Dress code, mixed sex provisions and the cultural environments at the facilities imposed restrictions on some people.’ **Rai and Finch (1997)** | Lack of culturally sensitive indoor facilities and services for both BME men and women. Facilities do not promote or incorporate BME religious and cultural practices |

**Supplemental Table S2 (Continued):** Cultural Expectations; key themes, second constructs and translations of one study into another.

| **Cultural Expectations; key themes** | **Extracted second order constructs**  **(authors’ own words or paraphrase)** | **Summary of translation across studies (Second order interpretation)** |
| --- | --- | --- |
| **Lack of time due to cultural obligations** | ‘Several men also commented on the time that they spent at their religious centre (mosque or temple).’ **Jepson *et al.* (2008)**  ‘Lack of time because of the demands of …..religious activities was seen to constrain opportunities for physical activity’ **Rai and Finch (1997)**  ‘Lack of time and opportunity was particularly a problem for female respondents, some of whom pointed to a cultural norm that once a woman got married, she was expected to stay indoors, attending to domestic chores and responsibilities’ **Lawton *et al.* (2006)**  ‘The girls are restricted to a certain time they have got to be in and lots of other things… There’s much more freedom for the guys.’ **Sportscotland (2001)** | The time constraints produced by competing cultural priorities limit participation in physical activity. The cultural obligations are religious and gender based with female members more likely affected. |
| **Language barriers** | ‘Unable to read or write their own language. This is not because the women are uneducated but rather that their education is an oral one. Consequently, South Asian Muslim women often feel uncomfortable at leisure centres where they may have difficulties with communication. They may be forced to rely on family or friends who speak English to act as interpreters. In the absence of such help, they would rather not encounter what is perceived as a hostile environment.’ **Carroll *et al.* (2002)**  ‘language is a key barrier to accessing health services’ **Farooqi *et al.* (2000)**  ‘Poor fluency in English, especially in the first generation, as a major barrier to accessing and understanding basic health information. One of the consequences was a reliance on other people, often family members, to access and interpret health information on their behalf. Poor English also limited people’s willingness to travel beyond the immediate neighbourhood (owing to difficulties in reading road names or asking directions).’ ‘Poor fluency in English played in constraining healthy lifestyle choices’ **Grace *et al.* (2008)**  ‘Some had experienced difficulties in communicating their needs. This was only a barrier to those for whom English was not their first language. Particularly where individuals felt unconfident, they were reluctant to risk being misunderstood or being embarrassed by not being able to fully understand the information provider. ‘‘This barrier was felt most strongly amongst older (first generation) individuals for whom English was not their first language.’ **Sportscotland (2001)** | Healthy lifestyle choices limited because of poor understanding or sharing of information about needs due to literacy problem. May be more likely among first generation or older immigrants. |

**Supplemental Table S2 (Continued): Cultural Expectations; key themes, second constructs and translations of one study into another.**

| **Cultural Expectations; key themes** | **Extracted second order constructs**  **(authors’ own words or paraphrase)** | **Summary of translation across studies (Second order interpretation)** |
| --- | --- | --- |
| **Gender issues** | ‘Expectation for women to remain in the home, dress modestly, and prioritise family and community over independence and social freedom’ **Grace *et al.* (2008)**  ‘Once a woman got married, she was expected to stay indoors, attending to domestic chores and responsibilities’ **Lawton *et al.* (2006)**  ‘Within the Indian community, this was an issue for married women only. For some, there was an expectation that the running the household and caring for the family should take precedence over and possibly exclude any extra-curricular activities’ **Sportscotland (2001)**  ‘This was intimately bound up with their identities as women—as homemakers, as carers of children and other relatives or in their other work…. Being active as a woman was a strong cultural obligation’ **Sriskantharajah and Kai (2007)** | Female expectations in South Asian community are higher compared to men as dictated by cultural norms and family obligations. |
