# Supplementary material for: Understanding the complex interplay of barriers to physical activity amongst black and minority ethnic groups in the United Kingdom: a qualitative synthesis using meta-ethnography
Source: BMC Public Health. 2015 Jul 12;15:643. doi: 10.1186/s12889-015-1893-0 (PMC4499183; doi:10.1186/s12889-015-1893-0)
Supplement: Additional files 3: Table S3. — Personal Barriers; key themes, second constructs and translations of one study into another. This table displays similar and opposite themes under the concept of ‘personal barriers” from across studies. The themes were translated into one another to produce second-order interpretation. This is a multipage table to be viewed as hyperlink. File exists in .txt format. [file 12889_2015_1893_MOESM3_ESM.doc]

**Supplemental Table 3: Personal Barriers; key themes, second constructs and translations of one study into another.**

| **Personal barriers; key themes** | **Extracted second order constructs**  **(authors’ own words or paraphrase)** | **Summary of translation across studies (Second order interpretation)** |
| --- | --- | --- |
| **Lack of time due to social obligations** | ‘Findings indicate that pressures to work long and/or antisocial hours, together with obligations to kin, may place particular demands and constraints on these people’s time’ **Lawton *et al.* (2006)**  ‘Women have generally increasing demands on their time, such as childcare’ **Carroll *et al.* (2002)**  ‘Prioritise family and community over independence and social freedom— for example, not to ask someone else to mind their children. Such norms potentially conflicted with efforts to achieve health related lifestyle change.’ **Grace *et al.* (2008)**  ‘Many families appeared to do little physical activity together, partly owing to the work commitments of the men. Many of the men worked long hours in shops or worked shifts as taxi drivers, which meant that they rarely had time off at weekends to spend with their wives and children.’ **Jepson *et al.* (2008)**  ‘following migration to the UK, long working hours, caring responsibilities’ **Netto *et al.* (2007)**  ‘Priorities were very much focused on establishing a secure and prosperous life within Scotland. These people tended to be working long hours to provide for their family, both on a daily basis and often at least six days a week.’ **Sportscotland (2001)**  ‘Women gave a low priority to physical activity as a leisure activity to be pursued in limited spare time when set against family and other obligations.**’ Sriskantharajah and Kai (2007)**  ‘Lack of time because of the demands of work, household management, childcare …..activities was seen to constrain opportunities for physical activity.**’Rai and Finch (1997)** | Time constraints due to priority of work commitments by male members of BME groups over physical activity to establish financial stability following migration. Pride in or priority of family commitment by female members of BME groups cannot be traded off. Difficulty to achieve independence and social freedom among BME groups. |

**Supplemental Table 3 (Continued):** Personal Barriers; key themes, second constructs and translations of one study into another.

| **Personal barriers; key themes** | **Extracted second order constructs**  **(authors’ own words or paraphrase)** | **Summary of translation across studies (Second order interpretation)** |
| --- | --- | --- |
| **Health problems** | ‘Health problems which could make physical activity difficult, these seemed to be reﬂected in, and reinforced by, their health beliefs; speciﬁcally, their perception that their diabetes weakened their bodies’ **Lawton *et al.* (2006)**  Respondents described how they had found the embodied experience of physical exertion unpleasant and sometimes even frightening, particularly when…, they felt that they were at risk of falling or fainting. **Lawton *et al.* (2006)**  ‘rather than seeing sweating, increased heart rate and breathlessness as ‘normal’ by-products of physical activity, some respondents perceived them as illness states and thus as something they should try to avoid’ **Lawton *et al.* (2006)**  Potential of harm threshold Fear of provoking harm or symptoms constraining physical activity is consistent with that found in other groups…. acknowledging any attendant anxiety, the potential exists to use this concept expressed in people’s own words (e.g. your body’s limit) to encourage raising their threshold by promoting activity and giving reassurance about safety. **Sriskantharajah and Kai (2007)**  Physical symptoms included chest pains, breathlessness, dizziness, drowsiness, body pains and fatigue **Sriskantharajah and Kai (2007)**  There is also awareness of the harm that can result from excessive or inappropriate exercise **Rai and Finch (1997)**  ‘reported their own ill-health, difficulty walking due to pain as a reason for no longer attending’ **William and Sultan (1999)** | Perceived adverse effects of physical activity is more prominent than benefits. Misperception that disease incompatible with physical activity. Physical activity is not considered as a disease modifier among BME groups as reinforced by health beliefs. Therefore, the fear of provoking physical symptoms rather than reported ill-health is a pronounced barrier in engaging in physical activity. |

**Supplemental Table 3 (Continued):** Personal Barriers; key themes, second constructs and translations of one study into another.

| **Personal barriers; key themes** | **Extracted second order constructs**  **(authors’ own words or paraphrase)** | **Summary of translation across studies (Second order interpretation)** |
| --- | --- | --- |
| **Lack of confidence** | lack of confidence in their ability to provide culturally relevant advice on lifestyle **Grace *et al.* (2008)**  The significance of a disruptive and disappointing migration experience, and how this can diminish the confidence of participants in their ability to explore outdoor environments. **Rishbeth (2004)**  ‘Individuals do not feel comfortable or confident participating alone. **Sportscotland (2001)**  Lack of confidence can also act as a barrier when trying sport for the first time. Some lacked confidence in their ability to communicate with others whilst playing sport. Lack the necessary skills to participate in this sport – thereby preventing some from even considering it as a possibility.**’ Sportscotland (2001)** | Lack of ability to negotiate or find neighbourhood facilities or use necessary skills for physical activity. Emerges from communication barriers, alien environment and lack of people to accompany to carry out physical activity. Associated generational effect in which first generation migrants with no social network are more prone to lack confidence. |
| **Lack of motivation** | A lack of motivation for exercise was described. Motivation was seen to be constrained by lacking will power, and not deriving enjoyment from physical activity **Rai and Finch (1997)**  ‘There appears to be reluctance to walk short distance’ **William and Sultan (1999)**  ‘lacking the time and motivation to undertake physical activity’ **Jepson *et al.* (2008)**  ‘Others described themselves as ‘lazy’ and as lacking the initiative to exercise on their own **Netto *et al.* (2007)** | No ‘will power’ or initiatives to engage in physical activity because there is no perceived enjoyment in physical activity. Another indication physical activity is perceived as a formal separate activity, rather than something to be done as part of everyday life. |
