# Supplementary material for: Understanding the complex interplay of barriers to physical activity amongst black and minority ethnic groups in the United Kingdom: a qualitative synthesis using meta-ethnography
Source: BMC Public Health. 2015 Jul 12;15:643. doi: 10.1186/s12889-015-1893-0 (PMC4499183; doi:10.1186/s12889-015-1893-0)
Supplement: Additional files 4: Table S4. — Factors limiting access; key themes, second constructs and translations of one study into another. This table displays similar and opposite themes under the concept of ‘factors limiting access” from across studies. The themes were translated into one another to produce second-order interpretation. This is a multipage table to be viewed as hyperlink. File exists in .txt format. [file 12889_2015_1893_MOESM4_ESM.doc]

**Supplemental Table 4: Factors limiting access; key themes, second constructs and translations of one study into another.**

| **Environmental factors**  **key themes** | **Extracted second order constructs**  **(authors’ own words or paraphrase)** | **Summary of translation across studies (Second order interpretation)** |
| --- | --- | --- |
| **Climate** | ‘dislike of going outdoors in bad weather in common with other cultural/ethnic groups’ **Lawton *et al.* (2006)**  ‘Further difficulties arose from poor climatic conditions, which were described as a major barrier to physical activity by men and women alike. Virtually all respondents expressed a profound dislike of going outside, particularly in cold, wet and windy conditions’ **Lawton *et al.* (2006)**  access to the outdoors in the UK and the weather and how this puts people off getting out and about’ **Jepson *et al.* (2008)**  ‘but walking is diﬃcult because of the climate’ **Keval (2009)**  ‘cold weather also cited as a reason preventing people from going out in the winter and participating in physical activity.’ **Rai and Finch (1997)**  ‘For some groups, weather, lack of public transport, and lack of knowledge about where to go where they would feel safe all are deterrents. **Sportscotland (2001)** | Attitudes to climate in the UK limit participation in outdoor activities. |
| **Distance** | The location (e.g. leisure centre) could prevent participation… Some reasons for choosing a location were dependent on variables such as access (e.g. distance from home) and would be similar for the population in the same geographical location. **Jepson *et al.* (2008)**  There was evidence that a lack of facilities in the area in which the individual lived was also a barrier for some. This meant that they were forced to travel longer distances (with cost implications) **Sportscotland (2001)**  Low levels of activity are related to lack of motivation alongside barriers such as poverty, transport, **Sriskantharajah and Kai (2007)**  ‘Some people felt there was lack of facilities in their locality’**’Rai and Finch (1997)**  ‘difficulty getting to group venue due to lack of transport’ **William and Sultan (1999)** | The lack of facilities or leisure centres in the locality is a barrier that implies increasing cost and time to travel longer distances from home. This is compounded by unfamiliar environment and cumulative cost in engaging in physical activity. |

**Supplemental Table 4 (Continued): Factors limiting access; key themes, second constructs and translations of one study into another.**

| **Environmental factors**  **key themes** | **Extracted second order constructs**  **(authors’ own words or paraphrase)** | **Summary of translation across studies (Second order interpretation)** |
| --- | --- | --- |
| **Lack of information** | ‘GPs’ surgeries do provide written translations of publicity material. However, this is redundant for those with literacy problems.’ **Carroll *et al.* (2002)**  ‘lack of informal support and access to essential services’ **Netto *et al.* (2007)**  **‘**There was less clarity about the type of physical activity that is most beneficial, about the duration, frequency and intensity of physical activity that is appropriate**.’ Rai and Finch (1997)**  ‘Individuals cannot even start to consider sport if they are not aware that it is an option available to them. At this stage, it is more about a general lack of awareness of facilities or activities available. That is, people are not actively seeking information at this point’ **Sportscotland (2001)**  ‘This lack of awareness also reinforced the perception that people from ethnic minority communities 'don't do sport'. **Sportscotland (2001)**  ‘Difficulties in accessing information about the nature and level of sporting activities available. This could be due to a number of reasons, but was often due to a lack of knowledge of where to look for information, even within people’s own community.’ **Sportscotland (2001)**  ‘Lack of confidence in gathering or seeking out information, given their ethnic origin. They either felt uncomfortable being in the minority, or were unconfident about asking for specific needs such as a women-only environment, difficulties in communicating their needs. This was only a barrier to those for whom English was not their first language’ **Sportscotland (2001).**  ‘Typically received cursory and general exhortations ‘to just do more exercise’ as part of other health consultations in primary and secondary care. Rather, they sought more detailed and speciﬁc guidance about appropriate exercise **Sriskantharajah and Kai (2007)**  ‘There was consistent uncertainty among women about what type and level of activity was appropriate and safe in relation to their illness. Advice from professionals to ‘just do exercise’ was inadequate. More effective guidance for targeted secondary prevention is needed at community and individual levels, reinforcing primary prevention and awareness raising. **Sriskantharajah and Kai (2007).** | Communication issues that include lack of awareness about facilities, benefits of physical activity and what constitutes physical activity. This is reinforced by poor information seeking behaviour among BME groups, language barriers. Inadequate personalised advice from health professionals about appropriate exercise for individual needs and illness. |

**Supplemental Table 4 (Continued):**  Factors limiting access; key themes, second constructs and translations of one study into another

| **Environmental factors**  **key themes** | **Extracted second order constructs**  **(authors’ own words or paraphrase)** | **Summary of translation across studies (Second order interpretation)** |
| --- | --- | --- |
| **Cost** | Access became more of a problem if transport had to be used, as this was seen as adding to the overall cost of exercising **Carroll *et al.* (2002)**  Practical barriers to a healthy lifestyle- lack of time or money **Grace *et al.* (2008)**  ‘People found the idea of spending money on physical activity alien compared with physical activity ‘back home’ which was integral to lifestyles and free. Spending money on physical activity could be regarded as ‘wasting money’ because it might otherwise be put to better and worthwhile uses. The idea of paying also had an effect in the restriction of opportunities for some people. **’Rai and Finch (1997)**  Migration to Britain and entrance into the lower echelons of economic society mean that many South Asian Muslim communities across Britain have been worst hit by economic decline. As discussed earlier, many such communities also live within economically deprived areas of cities. Consequently, a large number of South Asian.  Muslim women belong to the lower socioeconomic groups **Carroll *et al.* (2002)**  More than one member of a household is forced to survive on state benefits. The problem is exacerbated when a household has a large number of dependants. In such circumstances, the cost of exercising becomes a real barrier. The cost of exercise is cumulative **Carroll *et al.* (2002)**  The barrier was more of an issue for those on a low income. They tended to be people who had either been in Scotland for a shorter period of time or who were earning a fairly restricted income. Given the low priority on sport, it was sometimes felt that these costs could not be justified relative to other commitments. **Sportscotland (2001)**  Exercise of Prescription schemes entail charges that are not affordable by many of the households mentioned above. Thus the cumulative costs of exercising prove prohibitive for frequent and long-term adherence. **Carroll *et al.* (2002)** | Cost of exercise is cumulative and compounded by travel cost, belonging to lower socio-economical groups, and experience of free facilities in country of origin. Expenditure on exercise is probably considered wasteful and low priority in comparison to other commitments. |

**Supplemental Table 4 (Continued): Factors limiting access; key themes, second constructs and translations of one study into another.**

| **Environmental factors**  **key themes** | **Extracted second order constructs**  **(authors’ own words or paraphrase)** | **Summary of translation across studies (Second order interpretation)** |
| --- | --- | --- |
| **Lack of childcare facilities** | ‘Structural barriers to a healthy lifestyle…..difficulties with childcare’ **Grace *et al.* (2008)**  **‘**one of the main barriers to undertaking more physical activity was the lack of childcare facilities in leisure centres as they only catered to the under fives.’ **Jepson *et al.* (2008)**  ‘A few women mentioned that a lack of, or limited crèche facilities could affect women with regard to the times they could use facilities’ **Rai and Finch (1997).**  ‘Where childcare facilities were available, some women pointed to this as adding to the cost of exercising.’ **Carroll *et al.* (2002)** | Women’s participation in physical activity limited by childcare difficulties perceived from additional cost, unawareness of childcare facilities and efforts required to secure childcare space. |
| **Unfamiliar neighbourhood** | ‘For these female respondents, lack of familiarization with their local neighbourhood could engender feelings of vulnerability when they left their homes, which were compounded by difficulties speaking English.’ **Lawton *et al.* (2006)**  ‘Revealed a loss of detailed engagement with place, an absence of knowledge of both locality and natural history, and a break in norms of open space recreation.’ **Risbeth (2004)**  ‘They were also slightly fearful of being in unfamiliar spaces – particularly for those who might not speak fluent English because there was the fear of getting lost and not having the confidence to approach people for directions.’ **Jepson *et al.* (2008)** | Unfamiliar environment provokes fear of unsafe environment and has feelings of vulnerability among BME groups. Therefore, it is difficult to locate facilities. This is compounded by language problems and poor information seeking behaviour. |
| **Discontinuation of facilities** | ‘Frustration they experienced when they had found culturally competent services (some mainstream, others for South Asians) which only had short term funding. Thus they may have been involved in an activity which they enjoyed, but which subsequently ceased to be offered. **Jepson *et al.* (2008)**  ‘Outside influences, beyond an individual's immediate control, can also prevent the continuation of sporting behaviour. In these circumstances, the individual's commitment cannot be questioned**’ Sportscotland (2001)** | The short-termism of projects leads to discontinuation of services due to lack of fund. This barrier is beyond BME individual’s control. |

**Supplemental Table 4 (Continued):** Factors limiting access; key themes, second constructs and translations of one study into another.

| **Environmental factors**  **key themes** | **Extracted second order constructs**  **(authors’ own words or paraphrase)** | **Summary of translation across studies (Second order interpretation)** |
| --- | --- | --- |
| **Unclean environment** | ‘As a barrier to walking in the local area, or in the wider community. Some, because of their religious beliefs, did not like dogs (in Islam dogs are considered to be unclean), whilst others, similar probably to many living in their local area, did not like the thought that they, and/or their children, might be exercising in an area where there was dog excrement. **Jepson *et al.* (2008)**  ‘Dog dirt is seen as a deterrent.’ **Sportscotland (2001)** | Contaminated or polluted environment (e.g. dog waste), personal and religious beliefs that deter from outdoor activities. |
| **Restriction on use of private home** | ‘Some people were restricted from using their homes for certain forms of physical activity because of lack of space’ **Rai and Finch (1997)** | An indication that individuals not allowed to use their rented apartment for exercise due to lack of space or landlord restrictions. |
